# ANKRD49 promotes the invasion and metastasis of lung adenocarcinoma via a P38/ATF‐2 signalling pathway

**DOI:** 10.1111/jcmm.17464

**Published:** 2022-06-30

**Authors:** Yue‐hua Liu, Meng Yuan, Bai‐xue Xu, Rui Gao, Yu‐jie You, Zhi‐xin Wang, Yong‐cai Zhang, Min Guo, Zhao‐yang Chen, Bao‐feng Yu, Qi‐Wei Wang, Hai‐long Wang, Min Pang

**Affiliations:** ^1^ School of Basic Medicine, Basic Medical Science Center Shanxi Medical University Jinzhong China; ^2^ Xi'an Jiaotong University‐Affiliated Honghui Hospital Xi'an China; ^3^ Department of Pulmonary and Critical Care Medicine, The First Hospital Shanxi Medical University; Shanxi Province Key Laboratory of Respiratory Disease Taiyuan China; ^4^ Department of Cardiothoracic Surgery The First Hospital, Shanxi Medical University Taiyuan China; ^5^ Laboratory of Animal Center, Shanxi Key Laboratory of Experimental Animal Science and Animal Model of Human Disease Shanxi Medical University Taiyuan China; ^6^ Class ZT011907, The First Clinical Medical College, Shanxi Medical University Jinzhong China

**Keywords:** ANKRD49, ATF‐2, lung adenocarcinoma, matrix metalloproteinases, P38/MAPK

## Abstract

Lung adenocarcinoma (LUAD) is the most challenging neoplasm to treat in clinical practice. Ankyrin repeat domain 49 protein (ANKRD49) is highly expressed in several carcinomas; however, its pattern of expression and role in LUAD are not known. Tissue microarrays, immunohistochemistry, *χ* ^2^ test, Spearman correlation analysis, Kaplan–Meier, log‐rank test, and Cox's proportional hazard model were used to analyse the clinical cases. The effect of ANKRD49 on the LUAD was investigated using CCK‐8, clonal formation, would healing, transwell assays, and nude mice experiment. Expressions of ANKRD49 and its associated downstream protein molecules were verified by real‐time PCR, Western blot, immunohistochemistry, and/or immunofluorescence analyses. ANKRD49 expression was highly elevated in LUAD. The survival rate and Cox's modelling analysis indicated that there may be an independent prognostic indicator for LUAD patients. We also found that ANKRD49 promoted the invasion and migration in both in *in vitro* and *in vivo* assays, through upregulating matrix metalloproteinase (MMP)‐2 and MMP‐9 activities via the P38/ATF‐2 signalling pathway Our findings suggest that ANKRD49 is a latent biomarker for evaluating LUAD prognosis and promotes the metastasis of A549 cells *via* upregulation of MMP‐2 and MMP‐9 in a P38/ATF‐2 pathway‐dependent manner.

## 1 INTRODUCTION

Lung cancer (LC) is a life‐threatening malady due to its high incidence and mortality rates worldwide, with a 5‐year survival rate of approximately 15% (varying from 4 to 17%) as the lack of effective early diagnosis and its high metastatic potential.^1^, ^2^ Approximately 85% of all the diagnosed LC cases belong to non‐small‐cell lung cancer (NSCLC) which is primarily divided into lung squamous cell carcinoma (LUSC) and lung adenocarcinoma (LUAD).^3^ LUAD accounts for more than 40% of lung carcinoma, and it is the most common subtype of NSCLC and one of the most laborious to treat in a clinical trial.^4^ Therefore, the search of novel molecules involved in the development of NSCLC would help elucidate the molecular mechanism of metastasis and lead to development of efficient tumour therapeutic interventions.

Many genes and their encoded proteins participate in the initiation and development of LUAD, which is a multistep and multistage process.^5^ Among these proteins, the ankyrin repeat domain (ANKRD) is a 30 ~ 34 residue peptide that acts as a motif for protein–protein interactions.^6^ ANKRD 49 has four ankyrin repeat motifs, and it is known to participate in the development of diverse diseases, such as depression, malignant gliomas, and gastric cancer.^7^, ^8^, ^9^ However, the role and expression pattern of ANKRD49 in LUAD are no clear. While molecular profiling of LUAD using tissue expression microarray analysis or RNA‐sequencing^3^, ^10^ may facilitate the identification of novel biomarkers for LUND prognosis and treatment, we performed tissue microarray assays in the present study to evaluate the levels of ANKRD49 protein in LUAD tissues together with their case‐matched adjacent normal tissues for comparison reasons. The latent prognostic significance of ANKRD49 in LUAD patients was determined by Kaplan–Meier univariate and multivariate survival analyses. The effects of ANKRD49 and its underlying mechanism in the development of LUAD were also examined both *in vitro* by using an A549 LUAD cell line and *in vivo* by using a nude mice model.

## 2 MATERIALS AND METHODS

### 2.1 Human samples and tissue microarrays

Eighty LUAD cases (aged from 37 to 80 years, mean 59.5 years) were randomly selected for the current study, and their clinicopathological characteristics are recorded and summarized in Table S1, including sex, age, histological grade, tumour size, tumour‐node‐metastasis (TNM) stage, lymph node metastasis, distant metastasis, and differentiation grade. None of the patients received any previous treatments, and their tissues were collected, formalin‐fixed, paraffin‐embedded, and histologically examined by two independent pathologists to determine their disease development stages. All patients were followed up from the date of surgery for a period of 90 months unless mortality occurred earlier with July 2018 as the follow‐up deadline. These days up to the follow‐up deadline or the date of mortality were recorded to determine the survival time of the patients. Formal written and informed consents were obtained from the patients and clinicians for the use of the samples for research. The study protocols were also reviewed and approved by the Ethics Committee of the First Hospital, Shanxi Medical University (No. 2019pK042).

### 2.2 Cell line and construction of stable cell lines

A LUAD A549 cell line was obtained from the Cell Culture Center of the Chinese Academy of Medical Sciences (Beijing, China). The cells were cultured in a humidified chamber containing 5% CO_2_ at 37°C in McCoy's 5A medium (PYG0025; Boster Biological Technology, Wuhan, China) that was supplemented with 10% (v/v) fetal bovine serum (11011–8611; TIANHANG Biological Technology, Hangzhou, China).

Lentivirus vectors for ANKRD49 overexpression (LV5‐EGla‐GFP‐Puro‐ ANKRD49), ANKRD49 knockdown [LV3(H1/GFP&Puro)‐shANKRD49] and ATF‐2 knockdown [LV10N9(U6/m Cherry&Puro)‐shATF‐2] were purchased from the Genepharma Company (Shanghai, China). Stable ANKRD49‐overexpression or knockdown A549 cell lines were generated by infection with their corresponding lentivirus vectors and screened with 2.5 mg/ml puromycin for 1 week. The stable cells were named vector‐A549, ANKRD49 OE‐A549, shcontrol‐A549, ANKRD49 702KD‐A549, and ANKRD49 829KD‐A549. Overexpression of ANKRD49 or its knockdown was verified using real‐time PCR and Western blot analyses. To determine the role of ATF in the expression of ANKRD49, some stable ANKRD49‐overexpressing A549 cells were further infected with the ATF‐2 knockdown lentiviruses. The sequences used in the construction of these lentivirus vectors are as following: shANKRD49‐702: GCAAGGATACCCTAGAACTCC; shANKRD49‐829: GGAAGGCTGTACAAATTCTTC, and ATF‐2 shRNA: GCTATTCCTGCATCAATTA. A scramble sequence was also used as TTCTCCGAACGTGTCACGT.

### 2.3 Tissue microarray construction and immunohistochemistry

Lung carcinoma tissues and their paired normal lung tissues which were greater than 2 cm away from the tumour sites were collected from each of the patients. These tissues were paraffin‐embedded, formalin‐fixed, and their sections were stained and inspected by two independent pathologists using haematoxylin and eosin (H&E) staining. For high‐throughput molecular analysis of these tissues, a tissue microarray was constructed as described by us previously.^9^ Briefly, paraffin‐embedded sections were sliced 4‐μm‐thick and immunohistochemically stained using a rabbit anti‐human ANKRD49 primary antibody (25034‐1‐AP, ProteinTech, USA) at 1:100 dilution in an antibody dilution buffer.

To reveal the role of ANKRD49 in the development of lung adenocarcinoma, nude mice were injected with ANKRD49‐overexpressing A549 cells through their tail vein, and their lung tissues were collected and analysed by immunohistochemistry for the expressions of NKX2‐1 and Napsin A, which are markers of lung adenocarcinoma.^11^, ^12^ For immunohistochemistry assays, a two‐step protocol of immunological staining was performed as previously described by us.^13^ Tissue slides with paraffin sections were deparaffinized by xylene and ethanol. Endogenous peroxidase activity was blocked with 3% H_2_O_2_ for 15 min at room temperature. For antigen retrieval, the slides were heated in citrate buffer for 20 min. Nonspecific binding was blocked with 10% fetal bovine serum in PBS overnight at 4°C and incubated overnight at 4°C with rabbit anti‐NKX2‐1 (1:100, 5587811P138, Sino Biological Inc), rabbit anti‐Napsin A (1:500, 102,221‐T08; Boster Biological Technology), rabbit anti‐fibronectin (1:100, bs‐13455R, Bioss Biotechnology), rabbit anti‐ANKRD49 (1:200, 25,034‐1‐AP, ProteinTech, USA), rabbit anti‐MMP‐2 (1:100, bs‐4599R, Bioss Biotechnology, China), rabbit anti‐MMP‐9 (1:100 bs‐4599R, Bioss Biotechnology, China), rabbit anti‐p‐P38 (1:100, 4511, Cell Signalling Technology, USA), or rabbit anti‐p‐ATF‐2 (1:100, bsm‐52134R, Bioss Biotechnology, China) antibodies. Following washes with PBS, the sections were incubated for 1 h at room temperature with secondary antibodies diluted 1:200 in 5% BSA. Tissues were washed in PBS and visualized with a CX31‐LV320 platform for quantitative image analysis. The immunostaining intensities of ANKRD49, NKX2‐1, Napsin A, MMP‐2, p‐P38, and p‐ATF‐2 were evaluated in accordance with the published *H*‐score method^14^: The value of *H*‐score = (% unstained tumour cells × 0) + (% weakly stained tumour cells × 1) + (% moderately stained tumour cells × 2) + (% strongly stained tumour cells × 3). Scores of 0 (100% negative tumour cells) and 300 (100% strongly stained positive tumour cells) was the minimum and maximum *H*‐scores, respectively.

### 2.4 Proliferation assay

Cell proliferation was performed using a Cell Counting Kit‐8 (CCK‐8) purchased from Boster Biological Technology (AR1160), according to the manufacturer's protocol. Briefly, cells were seeded in triplicate at 2 × 10^3^ cells per well in 96‐well plates. 10 μl of CCK‐8 kit reagent was added to each well and incubated at 37°C for 2 h. The final absorbance (OD value) of the cell culture supernatants was measured at 490 nm with a microplate reader (SpectraMax®, 190 USA). Cell viability was calculated and expressed as the ratio of the OD490 values for vector‐A549, ANKRD49 OE‐A549, shcontrol‐A549, ANKRD49 702KD‐A549, or ANKRD49 829KD‐A549. The experiment was repeated three times in triplicate for each test group.

### 2.5 Colony formation assay

Vector‐A549, ANKRD49 OE‐A549, shcontrol‐A549, ANKRD49 702KD‐A549, or ANKRD49 829KD‐A549 cells (200 cells/well) were seeded in 6‐well plates in triplicate and cultured for 10 days. Dulbecco's phosphate‐buffered saline (DPBS, pH 7.4), Trypsin–EDTA (0.05%), Crystal Violet, ethanol (190 Proof), and 0.1% crystal violet solution were used. Count of colonies in control and treated cells was performed manually. Only colonies containing more than 50 cells (i.e., relatively large) should be counted.

### 2.6 Wound healing assay

The A549 cells were cultured in a 6‐well plate and used for wound healing assay. A straight line was scratched in each well of cells to wound the monolayer of cell growth using the tip of a sterile 1000‐μl‐pipette tip, and the cells were then washed with PBS. The cell cultures were maintained for 48 h in a humidified chamber containing 5% CO_2_ at 37°C in McCoy's 5A tissue culture medium supplemented with 1% fetal bovine serum. Images of wound healing were observed and recorded using an optical microscope, and the spatial distance between wound cells or the wound width was determined as follows: the transferred distance of 24 h (μm) = width at 24 h subtracts width at 0 h, the transferred distance of 48 h (μm) = width at 48 h subtracts width at 0 h. The experiment was repeated three times.

### 2.7 Cell migration and Matrigel invasion assays

For *in vitro* migration and Matrigel invasion assays, 5000 cells were seeded in the upper chamber of the transwells in 300 μl serum‐free tissue culture medium, and 600 μl medium containing 10% FBS was added to the lower chamber in which the FBS acted as a chemoattractant. For invasion assay, the upper chamber was pre‐coated with Matrigel (BD Biosciences, USA) to serve as a reconstituted basement membrane *in vitro*, occluding the pores of the membrane and blocking non‐invasive cells from migrating through the membrane. After incubation for 48 h, cells in the upper chamber were wiped off with a cotton swab, and the invaded cells to the underside of the membrane were fixed, stained with 0.1% crystal violet (8.0 μm, Costar, Corning, USA), and counted in five randomly selected microscopic fields (200×) per transwell membrane.

### 2.8 RNA extraction and real‐time PCR analysis

Total RNAs were collected using an RNA prep pure Tissue Kit (LS1040, Promega, Wisconsin, USA) according to the manufacturer's instructions. cDNAs were synthesized using an RT‐PCR Kit (Perfect Real Time) (MF166‐01, Mei5 Biotech, China). qPCR was performed with an ABI Step‐One Plus Real‐Time PCR System (Applied Biosystems, Thermo Fisher Scientific, USA) using the SYBR 2× M5 HiPer Real‐time PCR reagents (MF797‐01, Mei5 Biotech, China). The primer sequences used for quantitative real‐time RT‐PCR are detailed in Table S2. β‐actin was used as an internal control for the q‐PCR normalizations. The levels of gene expression were calculated using the comparative Ct method (2^−ΔΔC*t*^) method for data analysis.

### 2.9 Western blot

Total proteins were extracted in an SDS lysis buffer (50 mM Tris–HCl/pH 6.8, 10% glycerol, and 2% SDS) and were loaded onto a 10% SDS‐PAGE gel, separated, and then transferred onto polyvinylidene difluoride (PVDF) membranes. After blocking with 5% skim‐milk, the membranes were immunoblotted with a rabbit polyclonal or monoclonal antibodies for ANKRD49 (1:500, 25,034‐1‐AP, Protein Tech, USA), MMP‐2 (bs‐4599R), MMP‐9 (bs‐4599R), and p‐ATF‐2 (1:1000, bsm‐52134R, Bioss Biotechnology, China); P38 monoclonal antibody (8690), p‐P38 (4511), SAPK/JNK monoclonal antibody (9258P), and p‐JNK (1:1000, 4668P, Cell Signalling Technology, USA); ATF‐2 (1:1000, BS1022, BioWorld, USA); β‐actin monoclonal antibody (AP0060), GAPDH monoclonal antibody (AP0066), or LaminB (1:5000, AP6001, BioWorld, USA). After washing, membranes were incubated for 1 h with a HRP‐conjugated secondary goat anti‐rabbit (1:5000, BA1050) antibodies obtained from Boster Biological Technology, China and developed using the ECL Western Blot Analysis system (proteinsimple, Alphalmager MINI, USA). Image J software was used to analyse the grey value of protein bands, and the ratio between the grey value of the target protein band and the grey value of the internal reference band was used to represent the expression level of the target protein, and the protein expression level of the control group was set to 1.

### 2.10 Gelatin zymography

The enzyme activities of MMP‐2 and MMP‐9 were measured by gelatin zymography. Conditioned serum‐free media of A549 cell cultures for 48 h were collected, mixed with non‐reducing sample loading buffers, and electrophoresed on polyacrylamide gels containing 0.1% (w/v) gelatin. Following electrophoresis, the gels were washed at room temperature for 1 h with 2.5% (w/v) Triton X‐100 in PBS and then incubated at 37°C for 48 h in a Tris–HCl buffer (pH 7.6), containing 5 mM CaCl_2_ and 0.02% Brij‐35. The gels were finally stained with 0.25% (w/v) Coomassie Brilliant Blue in 40% (v/v) methanol and 7% (v/v) acetic acid. Proteolysis was detected as a clear white zone in a dark blue background. MMP‐9 and MMP‐2 were detected as bands at approximately 92 kDa and 72 kDa, respectively. Gelatinase activities for MMP‐9 and MMP‐2 reflected by the sizes of the clear zone in the gels and were captured and analysed using Image J software.

### 2.11 Cytoplasmic and nuclear protein extractions

Subcellular fractions of A549 cells were extracted using a Subcellular Proteome Extraction Kit (AR0106, Boster Biological Technology, USA) according to the manufacturer's protocols. Protein concentrations were measured by the Enhanced BCA Protein Assay Kit (P0012S, Beyotime Biotechnology, China), and 30 μg protein of each sample was used for Western blot analysis.

### 2.12 *In vivo* tumour xenograft assays

Ten female BALB/c nude mice (aged 4 ~ 6 weeks) were purchased from the Laboratory Animal Center of Shanxi Medical University (Shanxi, China) and were housed under specific pathogen‐free conditions (23°C, 60% relative humidity, a 12 h light–dark cycle, food, and water provided *ad libitum*). All experiments were approved by the Animal Ethics Committee of Shanxi Medical University (Permit no. SYDL2021036) and carried out according to the guidelines of the National Institutes of Health Guide for the Care and Use of Laboratory Animals (NIH publication no. 85–23, revised 1996). The mice were randomly divided into two groups (five mice per group), and either vector‐A549 or ANKRD49 OE‐A549 (4 × 10^6^cells/mice) was implanted subcutaneously into the lateral tail veins of the mice. After injections, the health of mice and tumour growth were monitored every day. Four weeks later, the mice were anaesthetised with sodium pentobarbital (75 mg/kg body weight, intraperitoneal injection) for collecting the lungs to count the metastatic nodules on their surfaces, and these tissues were then fixed in 4% paraformaldehyde for further haematoxylin and eosin staining or immunohistochemistry analysis.

### 2.13 Immunofluorescence

Cells (Vector‐A549, ANKRD49 OE‐A549) for immunofluorescence staining were grown and treated in chamber slides, fixed in 4% formaldehyde for 10 min, permeabilized for 10 min with 0.1% Triton X‐100 in PBS, and blocked with 5% BSA for 1 h. The primary antibodies used were diluted to 1:100 in 5% BSA/PBS and incubated for 1 h at room temperature. The secondary antibody was purchased from BOSTER (DyLight594 Conjugated AffiniPure), diluted to 1:100 in PBS, and incubated for 1 h. Images were captured with the Nikon microscope. Five visual fields were randomly selected, 100 cells were counted, and the percentage of positive cells in nuclear staining was calculated. All experiments were repeated three times.

### 2.14 Statistics

All statistical analyses were performed using the Statistical Package for the Social Science (SPSS) 20.0 software (SPSS Inc). Data are expressed as the means ± standard error of the mean (SEM). The Mann–Whitney *U*‐test was used for comparisons between the tumorous tissues and adjacent non‐tumorous, and Dunnett's *t*‐test was used to compare the differences between these groups. The *χ* ^2^ test and Spearman correlation analysis were used to examine the correlation between ANKRD49 protein expression and the clinicopathological parameters of the patients. Survival curves were established according to the method of Kaplan–Meier curve analysis, and they were compared by using the log‐rank test. Cox's proportional hazard modelling was used for multivariate analysis of the prognostic values of ANKRD49 in LUAD. A *p* value less than 0.05 was considered statistically significant in all statistical analyses.

## 3 RESULTS

### 3.1 ANKRD49 is highly expressed in LUAD and correlates with poor prognosis

**FIGURE 1:**
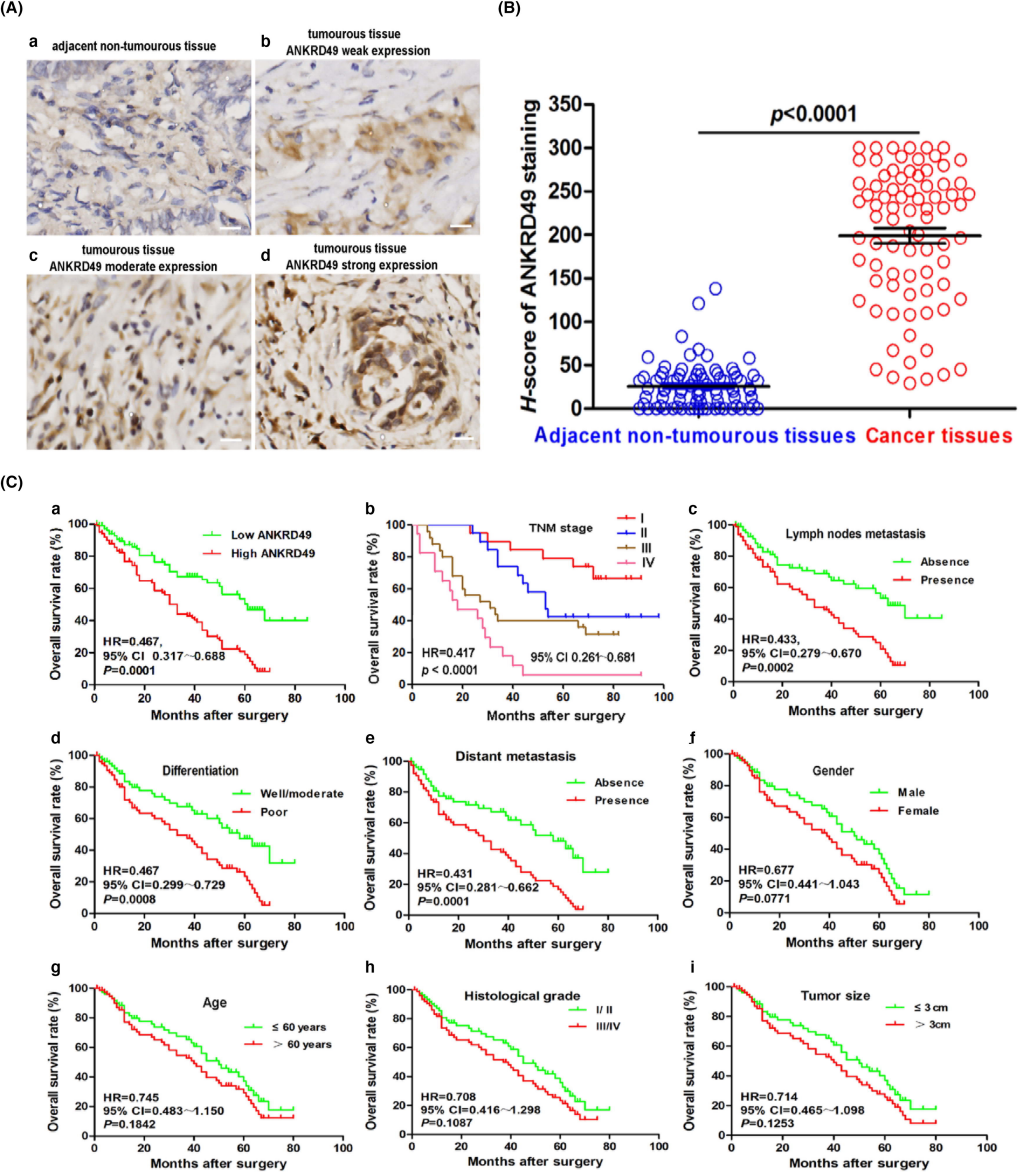
ANKRD49 is highly expressed in LUAD and correlates with poor prognosis. (A) Immunohistochemical determination of ANKRD49 in lung adenocarcinoma and its adjacent non‐cancerous tissues. Representative images are shown for negative ANKRD49 staining in adjacent non‐tumorous tissues (a) and weak ANKRD49 staining (b), moderate ANKRD49 staining, (b) and strong ANKRD49 staining (d) in tumour tissues. Scale bar is 50 μm. (B) Quantitative analysis of immunostaining intensities of ANKRD49 in lung adenocarcinoma tissues and their adjacent non‐tumorous tissues using the *H*‐score method. (C) Clinicopathological parameters associated with overall survival in lung adenocarcinoma based on Kaplan–Meier survival curves. These parameters include overall survival rate (a), TNM stage (b), lymph node metastasis (c), differentiation (d), distant metastasis (e), gender (f), age (g), histological grade (h), and tumour size (i). The horizontal axis represents overall survival time (months), and the vertical axis represents overall survival rate (%)

To determine the expression pattern of ANKRD49 in human lung adenocarcinoma tissues and their adjacent non‐tumorous tissues, immunohistochemistry was performed (Figure S1). As illustrated in Figure 1A, positive ANKRD49 immunoreactivities were detected in lung adenocarcinoma tissues (Figure 1Ab‐d), but not in their adjacent non‐tumorous tissues (Figure 1Aa). The median *H*‐score of ANKRD49 expression in lung adenocarcinoma tissues was 224.5 (29.0 ~ 300), and it was 22.0 (0 ~ 138) in the adjacent non‐tumorous tissues (Figure 1B). Therefore, the level of ANKRD49 expression was significantly higher in LUAD tissues than their adjacent non‐tumorous tissues (Figure 1B). When the relationship between ANKRD49 expression and clinicopathological parameters of LUAD was assessed, it was found that ANKRD49 expression was markedly higher in advanced stages of tumour development, including stages III and IV (*p* = 0.006) of the tumour, node, and metastasis (TNM) staging system for lung cancer, lymph node metastasis (*p* = 0.022), distant metastasis (*p* = 0.025), and differentiation (*p* = 0.006), compared to early stages of tumour development, including TNM stages I and II, and metastasis without lymph node (Table 1). No statistical differences were found between the levels of ANKRD49 expression and sexes (*p* = 0.102), ages (*p* = 0.214), histological grades (*p* = 0.185), or tumour sizes (*p* = 0.124) (Table 1). These data demonstrate that enhanced levels of ANKRD49 expression are closely associated with advanced stages of LUAD development, including TNM stage III and IV, lymph node metastasis, distant metastasis, and differentiation of the tumour, but not sex, age, histological grade, or tumour size.

**TABLE 1:** Correlation between ANKRD49 and clinicopathalogical characteristics in LUAD tissues

Clinical characteristics	Cases (*N* = 80)	ANKRD49 expression level		*χ* ^2^	*p* value	*r*
		High (*n*, %) (H‐score > 224)	Low (*n*, %) (H‐score ≤ 224)			
Gender						
Male	41	10 (24.4)	31 (75.6)	1.616	0.102	0.015
Female	39	9 (23.1)	30 (76.9)			
Age (years)						
≤60 years	41	7 (19.5)	34 (80.5)	0.626	0.214	0.013
>60 years	39	11 (28.2)	28 (71.8)			
Histological grade						
I/II	38	17 (44.7)	21 (55.3)	0.802	0.185	0.076
III/IV	42	23 (54.7)	19 (45.3)			
Tumour size						
<3 cm	30	5 (16.7)	25 (83.3)	1.330	0.124	0.042
≥3 cm	50	14 (28.0)	36 (72.0)			
TNM stage						
T1	19	6 (31.6)	13 68.4)	22.48	0.006^a^	0.617
T2	19	7 (36.8)	12 (63.2)			
T3	25	14 (56.0)	11 (44.0)			
T4	17	8 (47.1)	9 (52.9)			
Lymph node metastasis						
Absence	36	12 (33.3)	24 (66.7)	4.042	0.022^a^	0.385
Presence	37	21 (56.8)	16 (43.2)			
Miss	7					
Distant metastasis						
Absence	30	13 (43.3)	17 (56.7)	3.828	0.025^a^	0.592
Presence	34	23 (67.6)	11 (32.4)			
Miss	16					
Differentiation						
Well/moderate	35	11 (31.4)	24 (68.6)	6.285	0.006^a^	0.507
Poor	36	22 (61.1)	14 (38.9)			
Miss	9					

Abbreviations: LUAD, lung adenocarcinoma; TNM, tumour‐node‐metastasis.

^a^ Means statistical significance.

Spearman's interrelated analysis showed that positive expression of ANKRD49 correlated well with TNM stage, lymph node metastasis, distant metastasis, and differentiation at high correlation coefficients of 0.617, 0.385, 0.592, and 0.507, respectively (Table 1). When the survival curves were constructed using the Kaplan–Meier method to evaluate the clinical significance of ANKRD49 expression, we found that a high level of ANKRD49 expression correlated with poor prognosis in the LUAD patients. The overall survival rate of LUAD patients with high ANKRD49 (*H*‐score > 224.5) was significantly lower than in those patients with low ANKRD49 (*H*‐score ≤ 224.5) (*p* = 0.001, HR = 0.387, 95% CI 0.317 ~ 0.688) (Figure 1Ca). Our analysis also showed that low rates of overall survival were related to TNM stage (*p* < 0.0001, HR = 0.417, 95% CI 0.261 ~ 0.681), lymph node metastasis (*p* = 0.0002, HR = 0.433, 95% CI 0.279 ~ 0.670), and differentiation (*p* = 0.0008, HR = 0.467, 95% CI 0.299 ~ 0.729) of the patients with LUAD (Figure 1Cb‐d) but not to their distant metastasis, sex, age, tumour size, and histological grade (Figure 1Ce‐i). Further Multivariate Cox regression analyses suggested that ANKRD49 expression, TNM stage, lymph node metastasis, and differentiation were independent prognostic indicators for the overall survival of LUAD patients, indicating that ANKRD49 may be used as an independent predictor of prognosis evaluation for LUAD development (Table 2). Together, these results suggested that a high level of ANKRD49 expression is closely correlated with poor LUAD prognosis.

**TABLE 2:** Cox's proportional hazard model analysis of prosnostic factors in LUAD

Clinical parameters	Univariate analysis		Multivariate analysis	
	HR (95% CI)	*p‐*value	HR (95% CI)	*p‐*value
Gender (Male vs. Female)	0.579 (0.658–1.062)	0.473		
Age range (>60 vs. ≤60)	0.649 (0.601–1.364)	0.275		
Histological grade (III/IV vs. I/II)	1.693 (0.782–1.964)	0.126		
Tumour size (≥3 cm vs. <3 cm)	2.172 (1.275–2.693)	0.196		
Distant metastasis (presence vs. absence)	3.375 (1.418–3.596)	0.029*	2.639 (1.836–3.529)	0.058
TNM stage (III/IV vs. I/II)	3.152 (2.135–4.267)	<0.001*	2.637 (1.486–3.925)	<0.001*
Lymph node metastasis (presence vs. absence)	1.493 (0.534–2.418)	<0.001*	1.391 (0.474–2.267)	<0.001*
Differentiation (well/moderate vs. Poor)	1.674 (1.169–2.261)	<0.05*	1.941 (0.617–2.196)	<0.05*
ANKRD49 protein expression (>224.5 vs. ≤224.5)	3.164 (2.723–4.615)	<0.001*	2.715 (1.934–3.17)	<0.001*

Abbreviations: CI, confidence interval; HR, hazard ratio; LUAD, lung adenocarcinoma; TNM, tumour‐node‐metastasis.

* *p* < 0.05 was considered statistically significant.

### 3.2 ANKRD49 promotes migration and invasion of A549 cells

To further investigate the potential role of ANKRD49 in the development of LUAD, we prepared the Lv5‐EGla‐GFP/Puro‐ANKRD49 lentivirus to overexpress ANKRD49 (ANKRD49 OE) or LV3‐H1/GFP&Puro‐shANKRD49 lentivirus to knockdown ANKRD49 (ANKRD49 KD) in the A549 cells. Successful ANKRD49 overexpression or its knockdown (Figure S2A,B) in A549 cells was confirmed by real‐time PCR and Western blot analyses, and their stable overexpression or downregulation cell lines were thus created.

**FIGURE 2:**
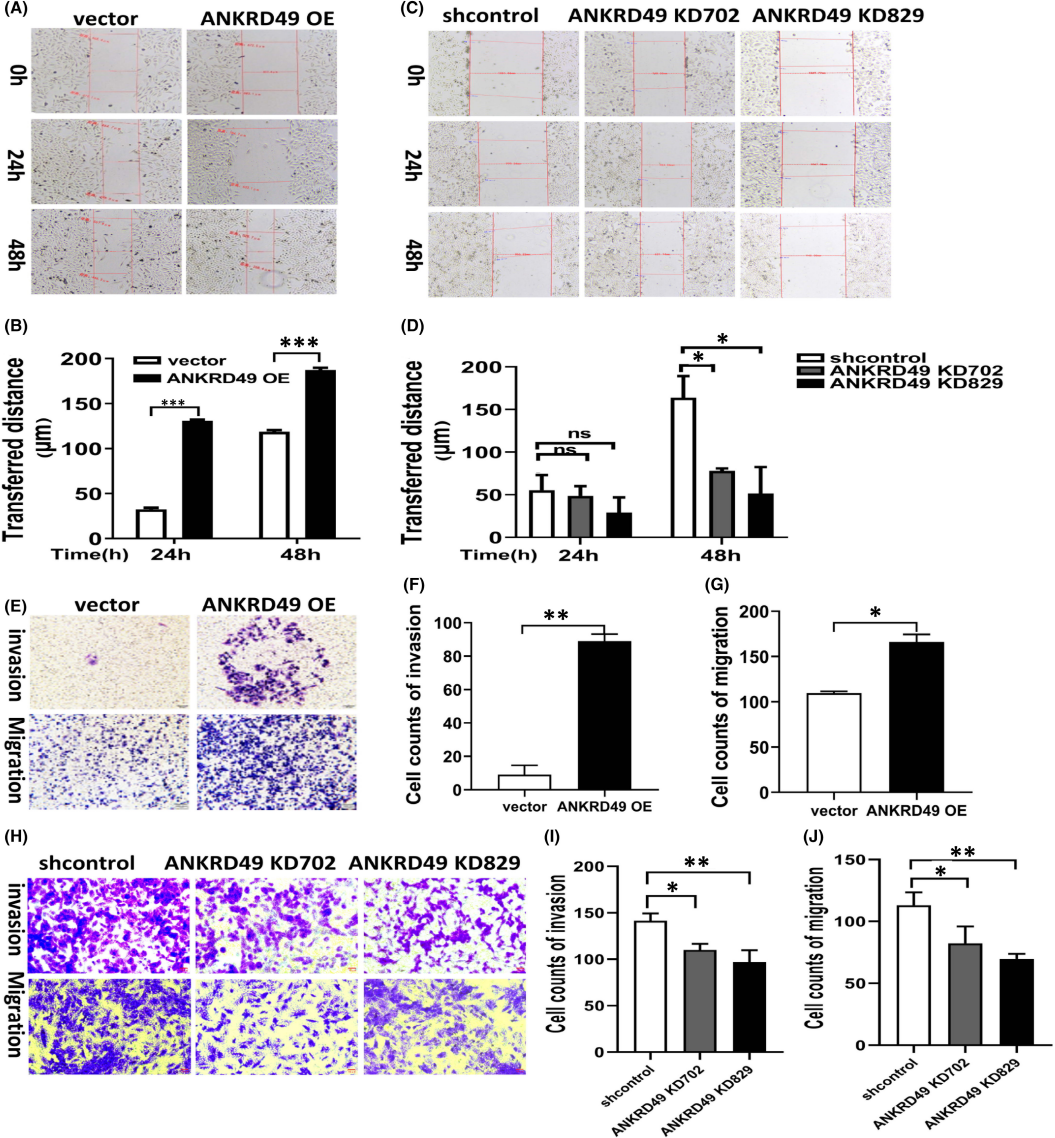
Effect of ANKRD49 overexpression and knockdown in A549 cells on cell migration and invasion. (A) Effect of ANKRD49 overexpression on A549 cell migration determined by scratch healing analysis after 24 h and 48 h; (B) Width of scratch wound after 24 h and 48 h. (C) Effect of ANKRD49 knockdown on A549 cell migration determined by scratch healing analysis after 48 h. (D) Width of scratch wound after 24 h and 48 h. (E) Matrigel invasion and migration assay of A549 cells following overexpression of ANKRD49 (ANKRD49 OE), compared to control cells (vector) in a 200× light by crystal violet staining. (F) Matrigel invasion analysis of ANKRD49 OE A549 cells or its vector control cells under a light scope in three randomly selected areas. (G) Transwell migration analysis of ANKRD49 OE A549 cells or its vector counterpart cells under a light scope in three randomly selected areas. (H) Matrigel invasion and migration assay of A549 cells after ANKRD49 knockdowns (ANKRD49 KD702 and ANKRD49 KD829), compared to their shcontrol cells by crystal violet staining. (I). Matrigel invasion analysis of A549 cells stably transfected with shcontrol, ANKRD49 KD702, or ANKRD49 KD829 under a light scope in three randomly selected areas. (J) Transwell migration analysis of A549 cells stably transfected with shcontrol, ANKRD49 KD702, or ANKRD49 KD829 vectors under a light scope in three randomly selected areas. Data are shown as mean ± SEM. *p < 0.05; **p < 0.01; ns, no significance

To assess the function of ANKRD49 on cell proliferation, CCK‐8 and colony formation assays were performed. The results showed that either ANKRD49 overexpression or its knockdown failed to proliferate A549 cells (Figure S2C–E) (*p* > 0.05). When wound healing and transwell assays were performed to evaluate the effects of ANKRD49 on cell invasion and migration, and we found that overexpression of ANKRD49 (ANKRD49 OE) increased the metastatic ability of A549 cells (Figure 2A,B, Figure 2E–G, *p* < 0.05). By contrast, knockdown of ANKRD49 substantially attenuated the metastatic ability of A549 (Figure 2C,D, Figure 2H–J, *p* < 0.05). These results demonstrated that ANKRD49 could promote cell migration and invasion in the LUAD A549 cells *in vitro*.

### 3.3 ANKRD49 enhances the invasion and metastasis of LUAD cells *in vivo*

**FIGURE 3:**
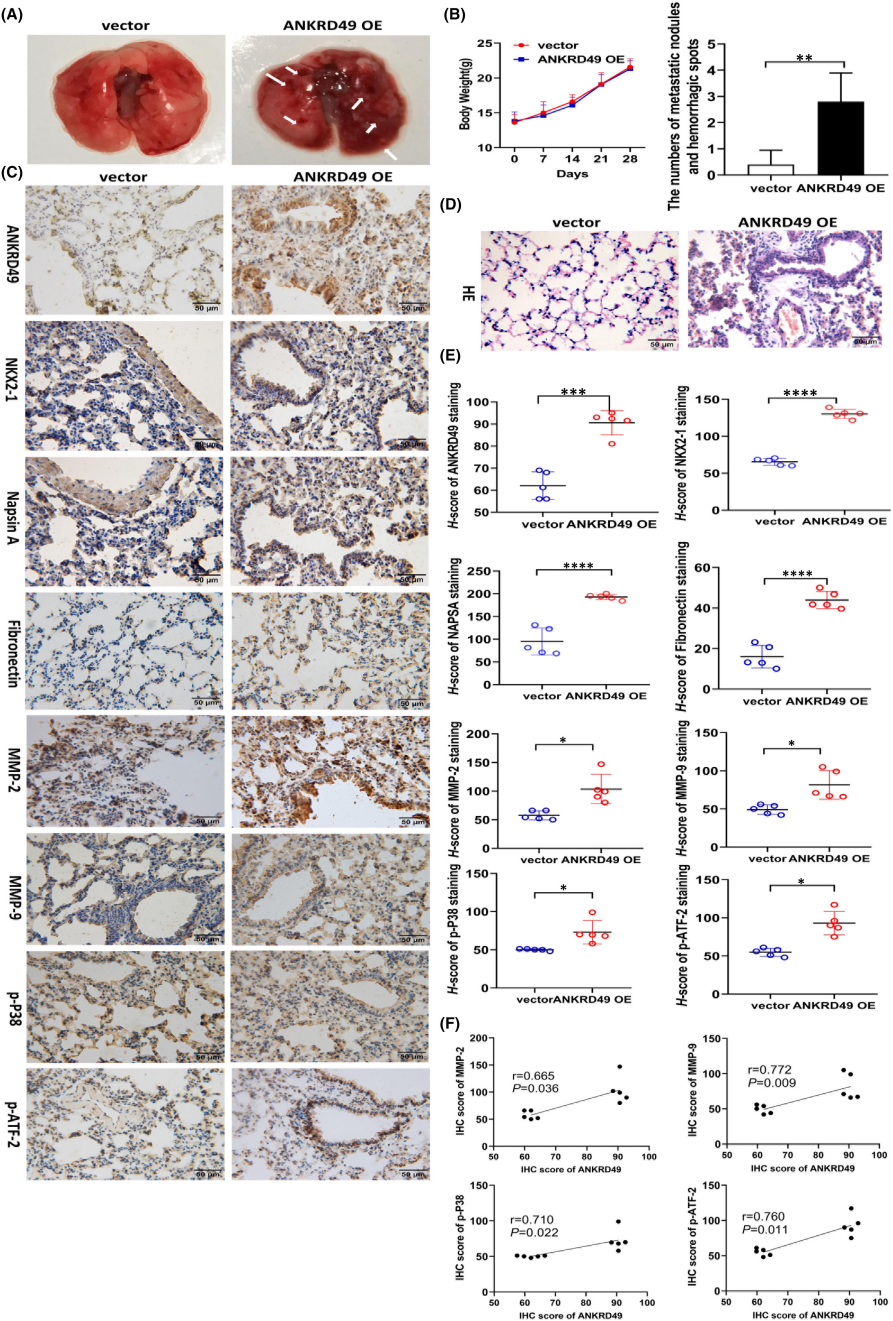
Effect of ANKRD49 expression on the invasion and metastasis and P38 MAPK/ATF‐2/MMPs signalling in nude mice in vivo. (A) Visible metastatic nodules and hemorrhagic spots on the lungs of the mice injected with ANKRD49 OE A549 cells but not their vector‐A549 cells. (B) Body weights and the number of metastatic nodules and hemorrhagic spots of the mice were determined following the injections of ANKRD49 OE A549 cells or their control vector cells (*n* = 5). (C) Immunoreactivities of ANKRD49, NKX2‐1, Napsin A, Fibronectin, MMP‐2, MMP‐9, p‐P38, and p‐ATF‐2 in lung tissues of the mice injected with ANKRD49 OE A549 cells or control vector‐A549 cells. Scale bar 50 μm. (D) HE staining of nude mouse lung tissue. Scale bar: 50 μm. (E) *H*‐score analysis of ANKRD49, NKX2‐1, Napsin A, Fibronectin, MMP‐2, MMP‐9, p‐P38, and p‐ATF‐2 immunoreactivities in lung tissues of the mice injected with ANKRD49 OE A549 cells or control vector‐A549 cells. (F) IHC score relevance of ANKRD49 and MMP‐2, MMP‐9, p‐P38, and p‐ATF‐2. Data are shown as mean ± SEM. *p < 0.05; **p < 0.01; ***p < 0.001; ****p < 0.0001; ns, no significance

To investigate the function of ANKRD49 in tumour invasion and metastasis *in vivo*, ANKRD49‐overexpressing (ANKRD49 OE) or its control vector‐A549 cells were injected into the lateral tail veins of nude mice (Figure S3). After 28 days following implantation, it was found that the numbers of metastatic nodules and hemorrhagic spots on the lungs of mice injected with ANKRD49 OE A549 cells were more than those in the mice injected with vector‐A549 cells (Figure 3A). HE staining of lung sections showed that the distribution of A549 tumour cells were mostly around the airway, and more cell invasions were seen in the ANKRD49 OE group (Figure 3D). The IHC assay also showed that ANKRD49 overexpression increased the immunoreactivities of NKX2‐1 and Napsin A, which were used to label the A549 cells injected into nude mice (Figure 3C,E). Fibronectin (FN) is involved in tumour invasion and metastasis. Tumour cells with high expression of FN on the cell surface can enhance the adhesion of tumour cells to each other and the anchoring adhesion ability of cells to matrix and basal membrane without shedding and metastasis through the action of various adhesion molecules on the cell membrane surface.^15^ We examined fibronectin expression in vector and ANKRD49 OE groups by immunohistochemistry (Figure 3D,E). We found that ANKRD49 overexpression increased the immunoreactivities of FN. Also, a higher level of ANKRD49 expression was also confirmed in the ANKRD49 OE mice group than their vector control mice group, as determined by IHC assay. Therefore, these data suggest that ANKRD49 can promote the invasion and metastasis of A549 cells in nude mice *in vivo*.

### 3.4 ANKRD49 promotes the expressions of MMP‐2 and MMP‐9

**FIGURE 4:**
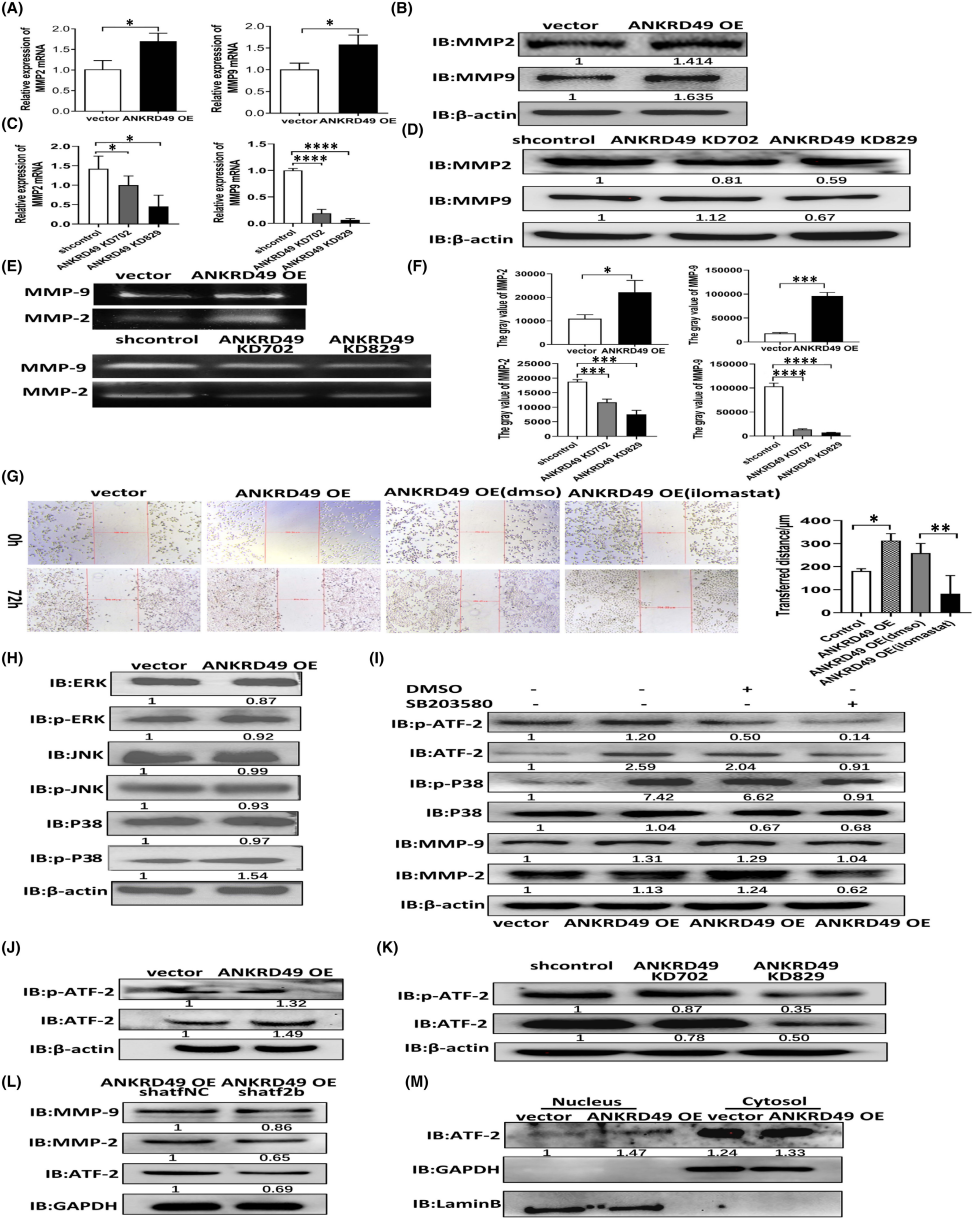
Effect of ANKRD49 on MMP‐2 and MMP‐9 expression, their enzyme activities, and the expression and activity of ATF‐2 transcription factor in A549 cells. (A) Expression levels of MMP‐2 and MMP‐9 mRNAs measured by q‐RT‐PCR in ANKRD49 OE A549 cells and their vector control cells. Data were calculated using 2^−ΔΔC*t*^ relative quantitative analysis. (B) Protein levels of MMP‐2 and MMP‐9 in ANKRD49 OE A549 cells or their vector control cells as determined by Western blot analysis. β‐Actin was used as the loading control. (C) Expression levels of MMP‐2 and MMP‐9 mRNAs were measured by q‐RT‐PCR in A549 cells stably transfected with ANKRD49 KD702, ANKRD49 KD829, and their shcontrol. Data were calculated using 2^−ΔΔCt^ relative quantitative analysis. (D) Protein levels of MMP‐2 and MMP‐9 in A549 cells stably transfected with ANKRD49 KD702, ANKRD49 KD829, and their shcontrol as determined by Western blot analysis. The knockdown effect of ANKRD49 and β‐Actin was measured using the same PVDF membrane and presented in Figure S2A. (E) Enzyme activities of MMP‐2 and MMP‐9 were detected by gelatin zymography. (F) Grey value of MMP‐2 and MMP‐9 in ANKRD49 OE A549 cells and ANKRD49 KD A549 cells. (G) Scratch healing analysis of ANKRD49 overexpression on A549 cell migration in the presence or absence of the MMP inhibitor (ilomastat) in 72 h. (H) Protein levels of ERK, p‐ERK, JNK, p‐JNK, P38 and p‐P38 determined in ANKRD49 OE A549 cells and their vector control cells by Western blot analysis. β‐Actin was used as loading control. (I) Levels of protein expression for p‐ATF‐2, ATF‐2, p‐P38, P38, MMP‐2 and MMP‐9 as determined by Western blot in ANKRD49 OE A549 cells and their vector control cells in the presence of a P38/MAPK inhibitor (SB203580) or its absence (DMSO). β‐Actin was used as loading control. (J) Protein levels of p‐ATF‐2 and ATF‐2 determined in ANKRD49 OE A549 cells and their vector control cells by Western blot analysis. (K) Protein levels of p‐ATF‐2 and ATF‐2 in A549 cells stably transfected with ANKRD49 KD702, ANKRD49 KD829, and their shcontrol as determined by Western blot analysis. β‐Actin was used as loading control. (L) Effect of ATF‐2 downregulation by RNA interference on MMP‐2 and MMP‐9 expression as determined by Western blot analysis in ANKRD49 OE A549 cells infected with lentivirus shatf2b or its vector control shatfNC. GAPDH was used as loading control. Data are shown as mean ± SEM. *p < 0.05, **p < 0.01, ***p < 0.0001, and ****p < 0.0001. (M) Protein levels of ATF‐2 in the cytoplasm and nucleus compartments of ANKRD49 OE A549 cells and their vector control cells as determined by Western blot analysis. GAPDH was used as cytoplasm loading control. LaminB was used as nucleus loading control

To explore the potential molecular mechanisms of ANKRD49 in LUAD invasion and migration, we examined some of the key players associated with tumour metastasis. The primary condition for invasion and metastasis of tumour cells is the degradation of extracellular matrix (ECM),^16^ which mainly depends on proteolytic enzymes, such as MMPs.^17^ MMPs play an important role in tissue remodelling and physiological homeostasis of extracellular matrix. Therefore, the gelatinolytic activities of MMP‐2 and MMP‐9 were determined by gelatin zymography (Figure 4E). We found that both MMP‐2 and MMP‐9 were significantly upregulated in ANKRD49 OE‐A549 cells compared to their control vector‐A549 cells (Figure 4A,B). When ANKRD49 OE‐A549 cells were pretreated with the MMP inhibitor ilomastat (at 10 μM),^18^ the migration and invasion of A549 cells were significantly inhibited as determined by wound healing assays (Figure 4G) (*p* < 0.05). Our next real‐time PCR and Western blot analysis further showed that overexpression of ANKRD49 in A549 cells promoted MMP‐2 and MMP‐9 expressions at both mRNA and protein levels. By contrast, knockdown of ANKRD49 in A549 cells was found to depress MMP‐2 and MMP‐9 expressions at both mRNA and protein levels (Figure 4C,D, *p* < 0.05). ANKRD49 enhanced expressions of MMP‐2 and MMP‐9 were also confirmed in immunohistochemistry experiments that demonstrated significantly high MMP‐2 and MMP‐9 immunoreactivities in the lung tissues obtained from nude mice injected with ANKRD49 OE‐A549 cells, compared to those from the mice injected with control vector‐A549 cells (Figure 3C,E) (*p* < 0.05). Our other available data suggested that the protein level of MMP‐2 (*r* = 0.665; *p* = 0.036) and MMP‐9 (*r* = 0.772; *p* = 0.009) is positively correlated with the level of ANKRD49 (Figure 3F). Taken together, these results demonstrate that ANKRD49 can promote the expressions of both MMP‐2 and MMP‐9 and their enzymatic activities in A549 cells.

### 3.5 ANKRD49 activates P38/MAPK pathway

Mitogen‐activated protein kinase (MAPK) plays an important role in cell division, differentiation, apoptosis, angiogenesis, and tumour metastasis. It promotes the occurrence and development of cancers by enhancing the survival and migration of tumour cells or their resistance to chemotherapy drugs through a series of cascade reactions involving ERK, JNK, and P38.^19^ As MAPKs are known to regulate the protein expression of MMP‐2 and MMP‐9,^20^ we therefore examined the expression of p38/MAPK, ERK, and JNK proteins in ANKRD49 OE‐A549 cells by Western blot analysis. We found that p‐P38/P38 expression was increased notably in ANKRD49 OE‐A549 cells but not the p‐ERK/ERK and p‐JNK/JNK proteins by ANKRD49 overexpression (Figure 4H). In addition, immunoreactivity of p‐P38 was also detected in the lung tissues of nude mice injected with ANKRD49 OE A549 cells but not the control vector‐A549 cells (Figure 3C,E). Our other available data suggested that the protein level of p‐P38 is positively correlated with the level of ANKRD49 (*r* = 0.710; *p* = 0.022) (Figure 3F).

To further demonstrate that P38 may mediate the increase of MMPs by ANKRD49 overexpression, ANKRD49 OE‐A549 cells were treated with a p38/MAPK inhibitor (10 μM, SB203580) or DMSO (10 μM) for 1 h. The results showed that administration of SB203580 significantly inhibited the expression of MMP‐2 and MMP‐9, compared to the DMSO vesicle control (Figure 4I). These findings suggested that P38/MAPK is required for ANKRD49‐mediated expressions of MMP‐2 and MMP‐9 activities in A549 cells.

### 3.6 ANKRD49 activates the ATF‐2 transcription factor

**FIGURE 5:**
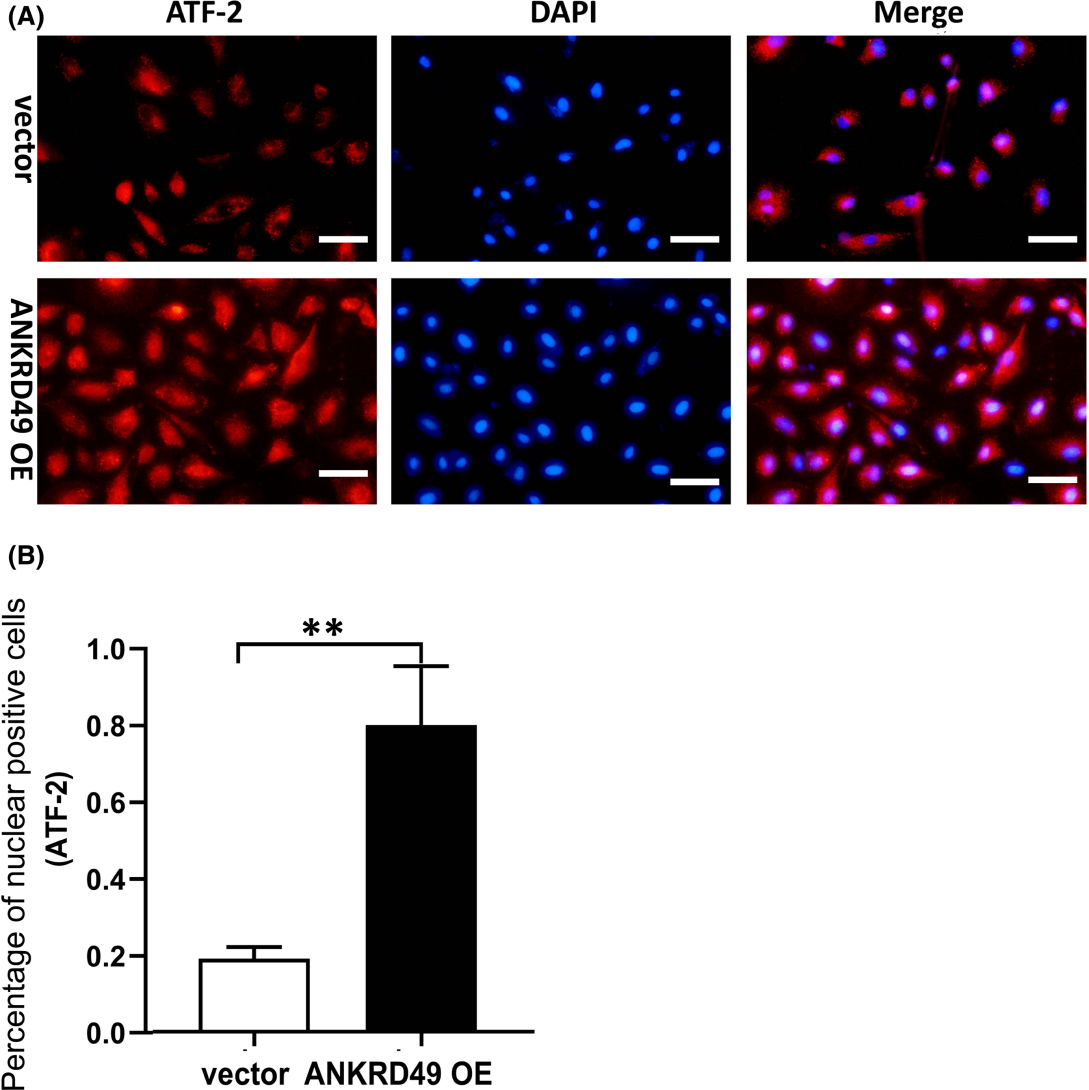
Immunofluorescence analysis of the nuclear distribution of ATF‐2 in vector‐ or ANKRD49 OE‐A549 cells. Representative immunofluorescence images (A) and percentage analysis of nuclear positive staining (B) are shown. Scar bar: 50 μm; Data are shown as mean ± SEM. **p < 0.01

Studies have shown that P38 phosphorylates and activates transcription factor 2 (ATF‐2),^21^ which participates in cell proliferation, invasion, and survival of cancer cells and plays an important role in tumorigenesis.^22^ Our results showed that the levels of ATF‐2 and p‐ATF‐2 were increased in A549 by ANKRD49 overexpression but reduced by ANKRD49 knockdown (Figure 4J,K). Interestingly, ATF‐2 and p‐ATF‐2 expression were also reduced by incubation of the ANKRD49 OE A549 cells with the p38/MAPK inhibitor, SB203580 (Figure 4I). Immunoreactivity of p‐ATF‐2 was also detected in the lung tissues of nude mice injected with ANKRD49 OE A549 cells but not the control vector‐A549 cells (Figure 3C,E). The available data suggested that the protein level of ANKRD49 was positively correlated with p‐ATF‐2 (*r* = 0760; *p* = 0.011) (Figure 3F). As nuclear translocation of ATF‐2, apart from its phosphorylation, is also involved in its transcriptional activity,^21^ we next measured the nuclear level of ATF‐2 in ANKRD49 OE A549 cells at the presence of KPT‐330, which is an inhibitor of nuclear export.^23^ We found that ANKRD49 enhanced the nuclear level of ATF‐2, compared to its vector controls (Figure 4M, Figure 5).

When ATF‐2 expression in ANKRD49 OE‐A549 cells was suppressed by RNA interference, we further found that downregulation of ATF‐2 markedly reduced the levels of MMP‐2 and MMP‐9, as determined by Western blot analysis (Figure 4I). These data together suggest that ANKRD49 can evaluate MMP‐2 and MMP‐9 via activating the ATF‐2 pathway.

## 4 DISCUSSION

LUAD accounts for approximately 40% of lung carcinoma cases, and it is responsible for approximately 1.6 million deaths worldwide.^24^, ^25^ Various treatment paradigms have emerged and been used for LUAD in the last few decades. These include multiportal or uniportal video‐assisted thoracoscopic surgery (VATS)^26^; immunotherapeutic agents, such as pembrolizumab and nivolumab^27^; targeted epidermal growth factor receptor (EGFR) mutations; and ALK gene rearrangement drugs, such as EGFR tyrosine kinase inhibitors (TKIs) and ALK inhibitors (erlotinib, afatinib, crizotinib, and ceritinib).^28^ Although significant progress has been made with these treatments, the overall 5‐year survival rate for LUAD remains considerably low at approximately 4–17%.^2^ A plethora of molecules that are highly expressed in LUAD have also been identified and considered diagnostic or prognostic biomarkers, such as serine protease inhibitor Kazal type 1 (SPINK1),^29^ C1q/tumour necrosis factor‐related protein 6 (C1QTNF6),^30^ and glucose‐6‐phosphate dehydrogenase (G6PD).^31^ These biomarkers still need to be further validated in clinical trials. Due to the heterogeneity of LUAD, it remains essential to discover more effective biomarkers to provide diagnostic, prognostic, or target therapeutic value in LUAD management.

The ankyrin repeat domain 49 protein (ANKRD49) contains four ankyrin repeat motifs, and it is closely associated with the occurrence and development of malignant gliomas and gastric cancer.^8^, ^9^ The ANKRD49 gene has also been detected as an invasion‐associated gene from microarray databases of the NCI‐60 lung cancer cell line.^32^ However, the clinical value and expression pattern of ANKRD49 in LUAD have not been fully characterized.

Our current study has detected the expression pattern of the ANKRD49 in LUAD and their paired normal lung tissues using a microarray panel and immunohistochemistry. A high level of ANKRD49 expression was revealed in LUAD tissues in contrast to their adjacent normal tissues, and the overall survival rate of LUAD patients with the high level of ANKRD49 expression was remarkably lower than those patients with low ANKRD40 expression. Our immunohistochemistry data further showed that high ANKRD49 expression was more easily visualized in tumours at advanced TNM stages (III‐IV), lymph node metastasis, and poor distal metastasis and differentiation than in tumours at early TNM stages (I‐II), lymph node‐lacking metastasis, or moderate distal metastasis and differentiation stages. Our Spearman correlation analysis further revealed a positive relationship between the level of ANKRD49 expression and advanced LUAD developments, including clinical TNM stage, lymph node metastasis, distal metastasis, and differentiation. These data demonstrate that the expression of ANKRD49 is closely associated with these clinicopathological parameters of LUAD and suggest that ANKRD49 participates in the invasion and metastasis of LUAD. Our Cox modelling also suggested that high level of ANKRD49 expression may be used as an independent prognostic factor for patients with LUAD and their rate of survival, in particular at advanced disease stages, including TNM stage (III‐IV), lymph node metastasis, and differentiation.

To further explore the role of ANKRD49 in LUAD, we employed a lung adenocarcinoma A549 cell line to create stable ANKRD49 overexpression or downregulated A549 cells. Our functional assays suggested that ANKRD49 can accelerate the invasion and migration of these A549 cells. When the stable ANKRD49 overexpression A549 cells were injected into nude mice, ANKRD49 was found to increase the numbers of metastatic nodules and hemorrhagic spots on the lungs of the mice *in vivo*. These results were in agreement with those data obtained from tissue microassay that ANKRD49 was associated with metastasis of lung adenocarcinoma.

Tumour invasion‐metastasis cascade is a multistep process where tumour invasion takes the first step for metastasis.^33^, ^34^ Extracellular matrix (ECM) participates in the formation of the tumour microenvironment in which tumour cells live. ECM degradation plays a key role in tumour invasion and metastasis. ECM degradation involves different types of proteases, but mainly the matrix metalloproteinases (MMPs), which belong to proteolytic enzyme subfamily and degrade almost all kinds of ECM proteins including connective tissues and basement membrane components.^35^ Among MMPs, MMP‐2 and MMP‐9 are mostly associated with ECM degradation and promote tumour cell invasion and metastasis.^16^ Herein, our results demonstrated that ANKRD49 not only boosts the level of MMP‐2 and MMP‐9 expression but also activates their proteolytic enzyme activities (Figure 4A–G).

Mitogen‐activated protein kinases (MAPKs), which consist of c‐Jun N‐terminal kinase (JNK), extracellular signal–regulated kinase (ERK), and P38 MAPK, regulate a wide range of cellular processes including gene expression, cell proliferation, cell movement, and apoptosis.^36^ We then assessed the effects of ANKRD49 on MAPKs and found that ANKRD49 enhanced the phosphorylation of P38 MAPK but not JNK and ERK. Previous studies have shown that P38 MAPK plays a central role in regulating the expressions of MMPs.^37^ Using a P38 MAPK inhibitor, we confirmed that ANKRD49 promoted the expression of MMP‐2 and MMP‐9 *via* P38 MAPK in this context. Next, we investigated how P38 MAPK affects the expression of MMP‐2 and MMP‐9. It is well‐established that P38 MAPK can phosphorylate and activate the activating transcription factor 2 (ATF‐2),^21^ which participates in proliferation, invasion, and survival of cancer cells and plays an important role in tumorigenesis.^22^ Our results illustrated that ANKRD49 raised ATF‐2 level and its phosphorylation (Figure 4J,K). ATF‐2 belongs to the activator (AP‐1) transcription factor family and interacts with other AP‐1 family members such as Jun to form homodimers or heterodimers that regulate the transcription of many genes, including MMP‐2 and MMP‐9.^38^, ^39^ Our knockdown of ATF‐2 assay also showed that ATF‐2 regulated the MMP‐2 and MMP‐9 expression. The form of Jun/ATF‐2 heterodimers helps stabilize nuclear localization of ATF‐2, while ATF‐2/ATF‐2 homodimers facilitates nuclear export of ATF‐2.^40^ We investigated the nuclear location of ATF‐2 in ANKRD49 overexpressed A549 cells but failed to detect ATF‐2. When the nuclear export inhibitor KPT‐330^23^ was present, we found that ANKRD49 increased the nuclear level of ATF‐2 (Figure 4M, Figure 5), suggesting a role of ANKRD49 in nuclear transportation of ATF‐2. Taken together, our data suggested that ANKRD49 activates P38 MAPK‐mediated expression of MMP‐2 and MMP‐9 in a manner of the ATF‐2‐dependent pathway.

Regulation of ATF‐2 activity depends on either its phosphorylation or its subcellular localization and stability.^41^ In this paper, we found that overexpression of ANKRD49 promoted the phosphorylation and nuclear location ATF‐2, indicating that ANKRD49 can enhance the activity of ATF‐2. Meanwhile, the level of ATF‐2 was also elevated by ANKRD49 over‐expression, suggesting that P38/MAPK may boost the expression of ATF‐2 directly or indirectly. It has been reported that PPARalpha, histone acetylation, and histone methylation are involved in the transcriptional regulation of ATF‐2.^42^, ^43^, ^44^ Additionally, several non‐coding RNAs (including microRNAs and lncRNAs can post‐transcriptionally modulate the expression of ATF‐2).^45^, ^46^ Therefore, further experiments are required to extensively explore the underlying molecular mechanisms of P38 MAPK on regulation of ATF‐2 activities.

In summary, this study demonstrates that ANKRD49 is highly expressed in LUAD patients and provides a powerful biomarker for use in the diagnosis and prognosis of LUAD in the clinics. Our results also show that ANKRD49 can promote the invasion and migration of A549 cells *via* a P38 MAPK/ATF‐2/MMPs signalling pathway *in vitro* and *in vivo*, suggesting a novel target for the remedy of lung adenocarcinoma. While this conclusion of the present study is based on limited sizes of the clinical samples, more clinical samples should be collected to further assess the correlation between the levels of ANKRD49 expression and the clinicopathologic features of LUAD patients. With the aids of much larger scale of tissue samples and more LUAD‐derived cell lines, further progress in establishing the role of ANKRD49 and its underlying molecular mechanisms in the development of LUAD would provide new targets for therapeutic intervention that might aid in the treatment of LUAD and related disorders.

## AUTHOR CONTRIBUTIONS

**Yuehua Liu:** Data curation (equal); validation (equal); writing – original draft (equal). **Meng Yuan:** Investigation (equal); validation (equal). **Baixue Xu:** Formal analysis (equal). **Rui Gao:** Visualization (equal). **Yujie You:** Validation (equal). **Zhixin Wang:** Investigation (equal). **Yongcai Zhang:** Resources (equal). **Min Guo:** Conceptualization (equal). **Zhaoyang Chen:** Investigation (equal). **Baofeng Yu:** Software (equal). **Qiwei Wang:** Visualization (equal). **Min Pang:** Conceptualization (equal); methodology (equal); writing – review and editing (equal).

## CONFLICT OF INTEREST

The authors declare no conflict of interest.

## Supporting information


Figure S1

Figure S2

Figure S3

Table S1‐S2

## Data Availability

Data available within the article or its supplementary materials.
