# Corrigendum

**DOI:** 10.1111/jcmm.17537

**Published:** 2022-10-10

In Figure 5A, vector group, the merged picture was not in accordance with ATF‐2 and DAPI staining, and it was a wrong cited picture. Herein, we provided a corrected merged picture for vector group in Figure 5A for replacing the wrong one in the published article by submitting a new submission according to the guide. 
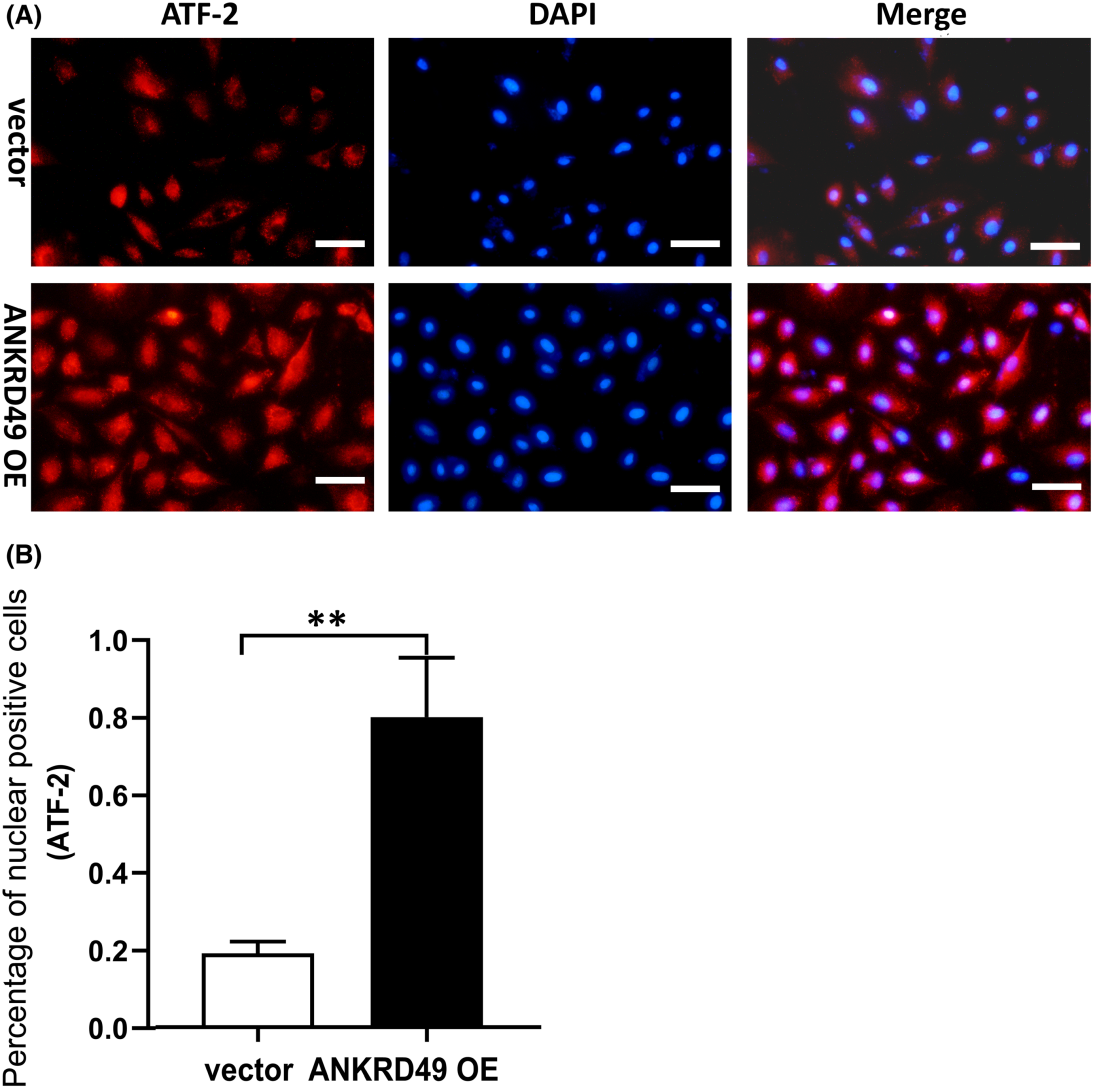


**FIGURE 5** Immunofluorescence analysis of the nuclear distribution of ATF‐2 in vector‐ or ANKRD49 OE‐A549 cells. Representative immunofluorescence images (A) and percentage analysis of nuclear positive staining (B) are shown. Scar bar: 50  μm; Data are shown as mean ± SEM. **p  <  0.01.
