# Supplementary figures and images for: ANKRD49 promotes the invasion and metastasis of lung adenocarcinoma via a P38/ATF‐2 signalling pathway
Source: J Cell Mol Med. 2022 Jun 30;26(16):4401–15. doi: 10.1111/jcmm.17464 (PMC9357638; doi:10.1111/jcmm.17464)

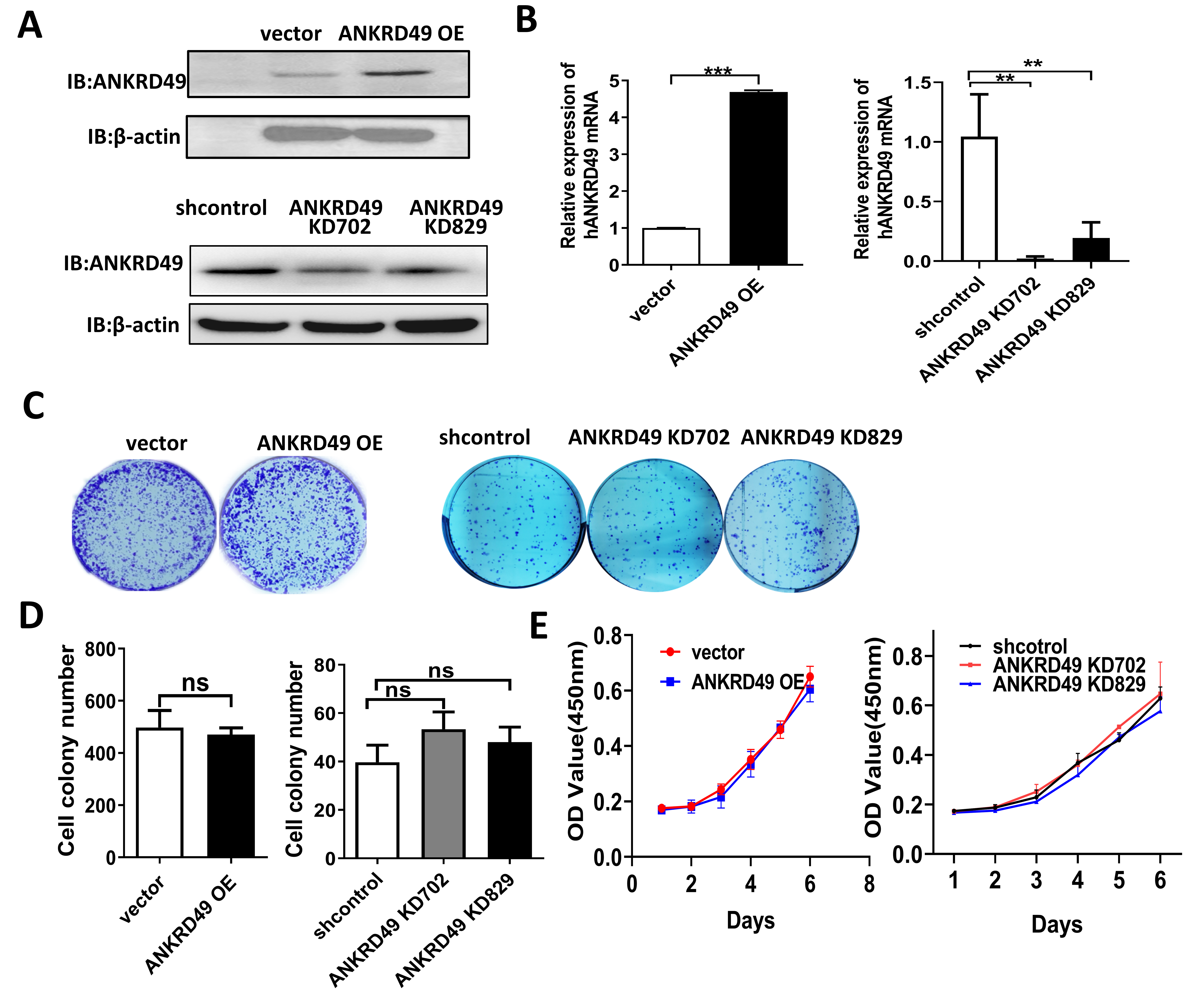

Supplement: Supplementary file 2 — Figure S2 [file JCMM-26-4401-s001.tif]

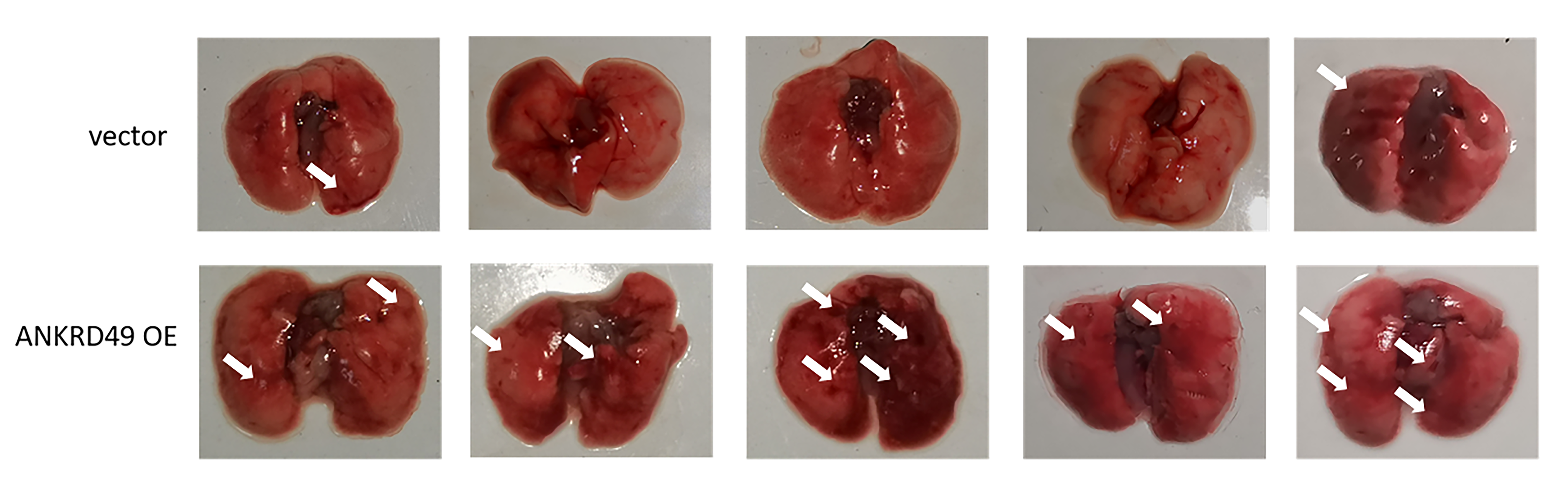

Supplement: Supplementary file 3 — Figure S3 [file JCMM-26-4401-s002.tif]
