# Supplementary material for: ANKRD49 promotes the invasion and metastasis of lung adenocarcinoma via a P38/ATF‐2 signalling pathway
Source: J Cell Mol Med. 2022 Jun 30;26(16):4401–15. doi: 10.1111/jcmm.17464 (PMC9357638; doi:10.1111/jcmm.17464)
Supplement: Supplementary file 4 — Table S1‐S2 [file JCMM-26-4401-s003.docx]

Supplemental table 1 Clinicopathalogical characteristics of LUAD cases

| Characteristic | Cases ( N=80) | % |
| --- | --- | --- |
| Gender | | |
| Male | 41 | 51.3 |
| Female | 39 | 48.7 |
| Age (years) | | |
| ≤60 years | 41 | 51.3 |
| >60 years | 39 | 48.7 |
| Histological grade | | |
| I/ II | 38 | 47.5 |
| III/IV | 42 | 52.5 |
| Tumor size | | |
| <3 cm | 30 | 37.5 |
| ≥3 cm | 50 | 62.5 |
| TNM stage | | |
| T1 | 19 | 23.8 |
| T2 | 19 | 23.8 |
| T3 | 25 | 31.3 |
| T4 | 17 | 21.1 |
| Lymph nodes metastasis | | |
| Absence | 36 | 45.0 |
| Presence | 37 | 46.3 |
| miss | 7 | 8.7 |
| Distant metastasis | | |
| Absence | 30 | 37.5 |
| Presence | 34 | 42.5 |
| miss | 16 | 20.0 |
| Differentiation | | |
| Well/moderate | 35 | 43.8 |
| Poor | 36 | 45.0 |
| Miss | 9 | 11.2 |

LUAD, lung adenocarcinoma; TNM, tumor-node-metastasis

Supplemental table 2 Primers used for quantitative real-time RT-PCR

| primer | 5’-3’ Forward | 5’-3’ Reverse |
| --- | --- | --- |
| ANKRD49 | TGGACACCTTATTCCTACTGG | AGTCTCCGCACTGTGGTAA |
| MMP-2 | TTGACGGTAAGGACGGACTC | GGCGTTCCCATACTTCACAC |
| MMP-9 | ACTACTCGGAAGACTTGCCG | AGGGACCACAACTCGTCATC |
| β-actin | CTGGCACCACACCTTCTACA | AGCACAGCCTGGATAGCAAC |
